# On the orientation of the chains in the mercerized cellulose

**DOI:** 10.1038/s41598-021-88040-x

**Published:** 2021-04-22

**Authors:** Dmitry V. Zlenko, Daria N. Vtyurina, Sergey V. Usachev, Aleksey A. Skoblin, Mariya G. Mikhaleva, Galina G. Politenkova, Sergey N. Nikolsky, Sergey V. Stovbun

**Affiliations:** 1 N.N. Semenov Federal Research Center for Chemical Physics RAS, Moscow, Russia; 2 Faculty of Biology, M.V. Lomonosov Moscow State University, Moscow, Russia

**Keywords:** Biopolymers, Supramolecular polymers

## Abstract

The cold alkaline treatment or mercerization of cellulose is widely used in industry to enrich the cellulose raw with high-molecular-weight $\alpha$-cellulose. Washing out of hemicelluloses by alkalies is accompanied by the rearrangement of the cellulose chains’ packing, well known as a transition between cellulose I and cellulose II. Cellulose II can also be produced by the precipitation of the cellulose solutions (regeneration). The currently accepted theory implies that in cellulose II, both mercerized and regenerated, the macromolecules are arranged antiparallelly. However, forming such a structure in the course of the mercerization seems to be significantly hindered, while it seems to be quite possible in the regeneration process. In this work, we discuss the sticking points in the theory on the antiparallel structure of mercerized cellulose from a theoretical point of view summarizing all of the available experimental data in the field.

## Introduction

Cellulose and chitin are common and wide-spread bio-polymers consisting of macroscopic fibers composed of smaller and mechanically stronger helical elements^1–3^. The smallest building block of the natural cellulose is an elementary fibril or nanofibril, a twisted bundle of parallel cellulose chains^2,4,5^. The lateral size of the nanofibril depends on the origin of the cellulose and ranges from 3–4 nm in softwood^6–9^, to 9 nm in cotton^10^, and up to 20 nm in cellulose from tunicates^5^. The nanofibrils, in their turn, twist into helical bundles known as microfibrils^11–13^ forming the complex hierarchy of chiral supramolecular structurallevels^14,15^. Biosynthesis provides for the co-directional (“parallel”) packing of the cellulose chains^16^. Accordingly, in the native cellulose I, the chains are oriented in parallel, and their directions coincide, which was confirmed by the numerous experimental works^4,17–19^ and usually considered to be well-proven.

It is well-known that cellulose swells and partially dissolves in aqueous solutions of sodium hydroxide at low temperatures^20–24^. The solubility of both cotton and bleached wood pulp in aqueous NaOH solutions with concentrations lower than 13% is rather small and does not exceed 30%^21^. The wood and cotton fiber of the sufficient degree of polymerization (DP) only swells in NaOH, and the fibers become ballooned in a very specific manner^23,24^. The solubility of cellulose in alkalies can be significantly increased by some additional components, such as zinc oxide^22^ or urea and its derivatives^24,25^. At the same time, a relatively low DP microcrystalline cellulose (MCC) almost completely dissolves in alkalies^21,26^, and such solutions remain stable at room temperature for weeks^26^. In the course of the swelling, the cellulose pulp elements pass apart from each other, the solvent-filled void space grows, and the terminal stage of this process would be complete dissolution. Therefore, the swelling can be considered as an initial stage of the dissolution.

Treatment of cellulose with alkali solutions causes not only its swelling but also mercerization, the process of conversion of the parallelly packed native cellulose I to cellulose II generally accepted to be antiparallelly packed^20,27–30^, similar to the early models proposed for cellulose I by Meyer^31,32^. If the change of the chains’ orientation occurs, it is most likely to occur at the very beginning of the mercerization process and the formation of the corresponding alcoholate (Na-cellulose I). The latter was confirmed by the decomposition of Na-cellulose I directly to cellulose II rather than cellulose I^27^. The tight relationship between the swelling and mercerization was indirectly demonstrated by hampered swelling and mercerization of highly crystalline cellulose I from *Valonia ventricosa*^33^.

Interpretation of the X-ray diffraction experiments^28–30^ seems to contradict some of the results obtained through other methods. For example, in mercerized cotton linter, silver staining of the reducing ends of the cellulose chains always took place only at one of the ends of the microcrystallites, which indicates the parallel orientation of the chains both in natural and mercerized cellulose^34^. Similarly, in the mercerized ramie, the silver labeling was observed at both ends of the bundles of cellulose microcrystallites (CNCs), but not the individual nanofibrils^35^. This observation allows explaining the antiparallel packing by the mutual interpenetrating of the cellulose chains from the different microrystallites^35,36^. The same was directly shown for cellulose I CNC through the chemical functionalization and aggregation^37^. However, the newer works dedicated to the aggregation of the CNC antecedently imply the antiparallel packing of the chains in cellulose II, while no evidence was provided^37,38^.

Molecular modeling of the packing of cellulose chains showed parallelly stacked chains could realize the packing with the unit cell parameters very close to those observed in cellulose II^39^. However, this model was not further discussed by authors^40^ and was not confirmed by alternative molecular modeling research^41^. However, Kroon-Batenburg and Kroon^40^ has shown the stability of the parallelly-packed structure of cellulose having the X-ray pattern similar to one of cellulose II and registered a spontaneous transition between this parallel packing and cellulose I$\beta$. The latter observation seems to be of particular importance and demonstrates the principal possibility of such an interconversion without any global rearrangement of the structure of microcrystallites. However, later the parallel-packed structure of cellulose II was additionally examined and rejected as a worse correlated with the experimental data^29,30^. The parallel packing of the chains in mercerized cellulose II was supported by the experiments on the generation of the second harmonic, which amplitude appeared to be much higher than expected based on the concept of antiparallel packing of the chains^42^.

In the course of the dissolution, the native supramolecular structure of cellulose breaks down, and no signs of the native cellulose nanofibrils or CNC can be found in the structure of the fibers or films made of the regenerated cellulose^43,44^. Accordingly, no data on the silver staining of the CNC ends can be obtained in the case of the regenerated cellulose. On the other hand, the diffractograms of the mercerized and regenerated cellulose II are a bit different^30,45^. Therefore, the structure of the regenerated and mercerized cellulose II can (but not necessary) be different. The significant differences in the spatial structure of the native cellulose pulp and cellulose solution make the latter proposition quite reasonable.

Despite the apparent differences in the structure of the natural and mercerized celluloses, the chains’ antiparallel orientation following from the X-ray patterns interpretation contradicts several results obtained earlier by other methods and freely available in scientific literature. However, the scientific society definitely considers cellulose II to be antiparallel despite the way it was obtained, either mercerization or regeneration. Here, we offer some additional theoretical reasoning against the chains’ antiparallel packing in the mercerized cellulose. Meanwhile, our reasoning does not concern the regenerated cellulose II, where the antiparallel packing does not contradict any experimental results and our theoretical derivations. Moreover, the difference in the structure of the mercerized and regenerated celluloses’ was directly indicated by the differences in their X-ray patterns^30,45^.

## Methods

### Feedstock

In this work, we have used cellulose raw of various origins. Bleached hardwood cellulose was supplied by Arkhangelsk paper and pulp mill (Russia). Cotton linter cellulose was supplied by “OOO Fargona Kimyo Zavodi” (Uzbekistan). Linnen cellulose was prepared from the flax straw (Tver region, harvested in 2019). After the mechanical sorting, the straw was treated with a 5% NaOH solution for 2 h at 93–95 °C (solvent-to-pulp ratio 50). After the washing until the neutral pH, the cellulose was bleached by a 1% $\hbox {H}_2\hbox {O}_2$ solution (1 h, 93–95 °C, solvent-to-pulp ratio 50). Then the cellulose was rewashed and dried at 20–22 °C for 72 h (the residual humidity was 5–7%).

As a method for increasing the share of alpha-cellulose, mercerization is most applicable to the wood pulp, which is usually enriched with the low-molecular-weight fractions. That is why, the majority of the results were explicitly presented only for wood cellulose. Results for other raw types were presented if only they differed from those obtained on the wood raw.

### Mercerization

Cellulose was treated with a 17.5% NaOH solution (solvent-to-pulp ratio 40) at a temperature of 22–22 °C for 40 min. Then the alkali was removed by washing in distilled water until the neutral pH. The washed samples were dried at 55 °C to the constant weight. Before analysis, the samples were kept at the constant relative humidity of $60\pm 5\%$, and a temperature of 20–22 °C for 72 h.

### X-ray diffractometry

The samples of the cellulose were not additionally treated before the X-ray examination. The samples’ moisture content was in the range of 5–7%. Wide-angle X-ray scattering experiments were made using two different sources. The first was the “DIXI” set-up at NIC Kurchatovsky Institute (Moscow, Russia), providing the synchrotron radiation with $\lambda = 0.69\,\AA {}$. The distance between the sample and the detector (CCD165 by Mar, USA) was 500 mm (transmitted radiation was detected). The second source was DRON-3 X-ray diffractometer (Russia), providing the X-ray radiation of $\lambda = 1.54\,\AA {}$. The DRON-3 diffractometer was equipped with a scintillation detector used for the reflected radiation analysis. Small-angle X-ray scattering experiments were also made at NIC Kurchatovsky Institute (Moscow, Russia) using the “Bio-MUR” set-up: $\lambda = 1.45\,\AA {}$), the distance between sample and detector (PILATUS 3M by Dectris, Switzerland) was 2200 mm (transmitted radiation was detected). The SAXS curves were approximated using the model of infinite cylinders.

### IR spectroscopy

The IR spectra were recorded using a Tensor 27 spectrometer (Bruker) with an ATR (Pike MIRacle) unit on a germanium crystal (resolution was 4 cm^−1^). Spectra were recorded using a Bruker Tensor 27 IR Fourier spectrometer with an ATR attachment with a fixed angle of reflection (Pike MIRacle). The resolution was $4\,\hbox {cm}^{-1}$.

### Optical microscopy

The diameter of the cellulose fibers immersed either in water or in the NaOH solution was measured using Leica DMI 6000 optical microscope. Five independent samples were analyzed per each time-point, and 200–300 cellulose fibers were measured in each sample. Each fiber was measured along its entire length with a step of $\sim 100\,{\upmu }\hbox {m}$.

## Results

### Mercerization

Treatment of cellulose with a NaOH solution (17.5%) leads to a partial dissolution and leaching of low-molecular-weight fractions from the fibers^20–22^. The mass share of high molecular weight $\alpha$-cellulose in the feedstocks was $96\pm 3$, $84\pm 3$, and $90\pm 2\%$ for cotton linter, hardwood and flax pulp, respectively^44^. Following mercerization, the share of $\alpha$-cellulose in all samples increased to 97–98%. The degree of polymerization (DP) in the studied samples did not change significantly during the mercerization process. It amounted to 1250–1500 and 1400–1650 for hardwood and linter cellulose, correspondingly, while DP of the flax cellulose was substantially higher than 10,000^44^.

**Figure 1:**
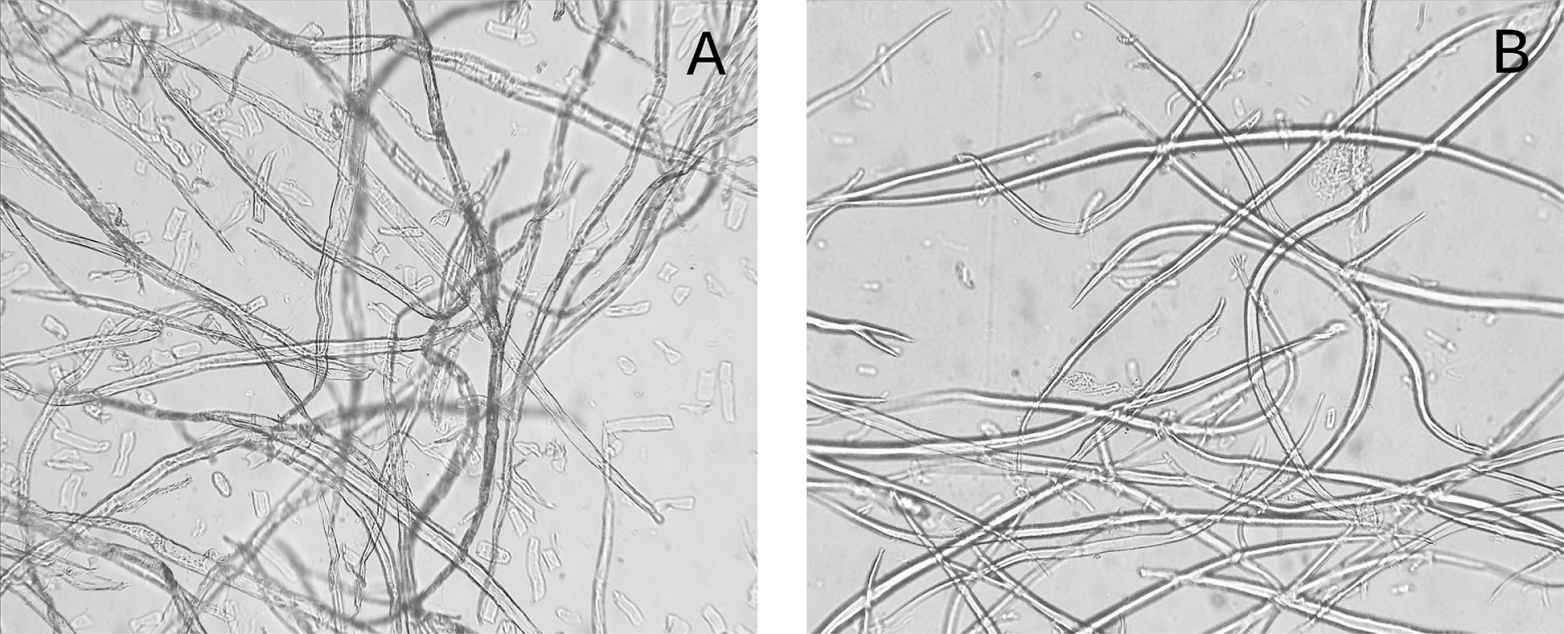
The optical micrographs of the hardwood pulp immersed in the distilled water (**A**) and 17.5% NaOH solution. Samples were incubated for 40 min before the examination.

### Cellulose swelling

Mercerization leads to slight changes in the external morphology of the fibers, which become smoother (Fig. 1), and also grew in diameter. We did not observe the balloons’ formation^23^; however, the low molecular weight fractions were dissolved and washed out during the treatment, which follows from increase of the $\alpha$-cellulose content. In the aqueous suspension, an average diameter of the hardwood pulp’s tracheids and libriform fibers was $46.8\pm 0.7$ ${\upmu }\hbox {m}$ ($\alpha = 0.05$). In comparison, in the alkaline solution, the fibers swelled to the average diameter of $51.5\pm 0.8\,{\upmu }\hbox {m}$ ($\alpha = 0.05$). The ratio of fiber diameters of the swollen and initial cellulose was $\sim 1.10$. Such a minor increase of the diameter allows proposing that the swelling process preserves the geometrical similarity between the initial and swollen fibers. The proposition made allows estimating the relative increase in cellulose volume during swelling in alkali as $\sim 1.10^3 = 1.33$. After washing out of alkali, the average diameter of the hardwood fibers became equal to $49.1\pm 0.9\,{\upmu }\hbox {m}$ ($\alpha = 0.05$), which gives a ratio of fiber diameters before and after mercerization of $\sim 1.05$. Using the same proposition on the geometrical similarity between the initial and mercerized fibers, the volumes’ ratio would be $\sim 1.05^3 = 1.15$. The obtained estimate is significantly lower than it was reported earlier^20,23^.

**Figure 2:**
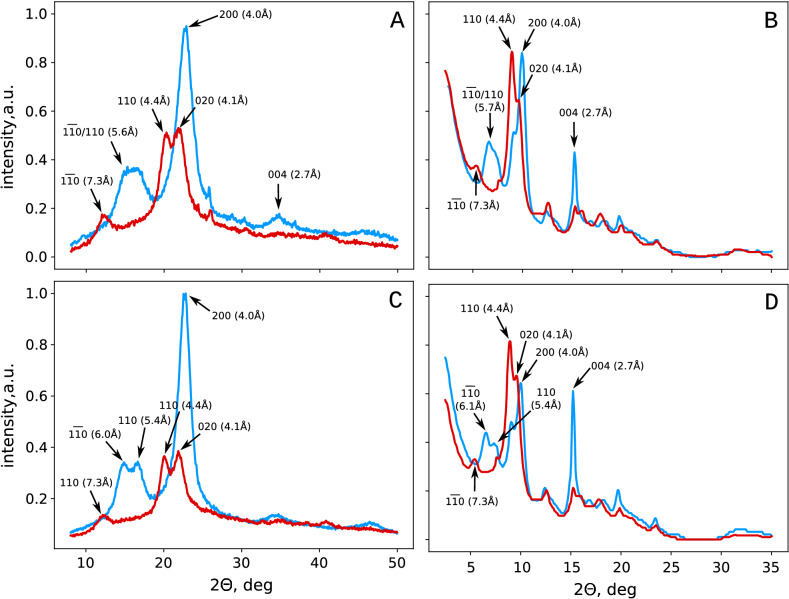
Typical diffractograms of the initial (blue curves) and mercerized (red curves) hardwood (**A**,**B**) and flax (**C**,**D**) cellulose obtained using the plain X-ray of $1.54\,\AA {}$ in reflection mode (**A**,**C**), or synchrotron X-ray radiation of $0.69\,\AA {}$ (**B**,**D**) in transmission mode. The arrows denote the prominent peaks attributed according to French (2014)^46^.

### X-ray diffractograms

Treatment with a strong alkali solution causes well-characterized changes in the wide-angle X-ray scattering (WAXS) patterns (Fig. 2), known as transitioning from cellulose I to cellulose II^28–30,46^. We got WAXS spectra for two wavelengths: plain X-ray of $1.54\,\AA {}$ in the reflection mode ((Fig. 2A,C) and $0.69\,\AA {}$) synchrotron radiation in transmission mode (Fig. 2B,D). In general, the observed spectra coincided. In the untreated hardwood cellulose, the broad double peak $(1{\bar{1}}0)/(110)$ was observed at $\Theta \sim 15$–$17\,^\circ$ ($\lambda = 1.54\,\AA {}$) that corresponded to the interplane distance of about $5.6\,\AA {}$ (Fig. 2A). In the flax cellulose I (Fig. 2C), this peak was resolved and corresponded to the interplane distances of 5.4 and $6.0\,\AA {}$. In the mercerized cellulose, peak $(1{\bar{1}}0)$ shifted to 7.3Å, while peak (110) shifted to $4.4\,\AA {}$ in hardwood and flax cellulose. The main characteristic peak of cellulose I (200) was not presented in the diffractogram of cellulose II, as well as peak (004), while peak (020) was clearly visible. The obtained pattern corresponds to the described earlier one that confirms the transition from cellulose I to cellulose II upon alkaline treatment.

**Figure 3:**
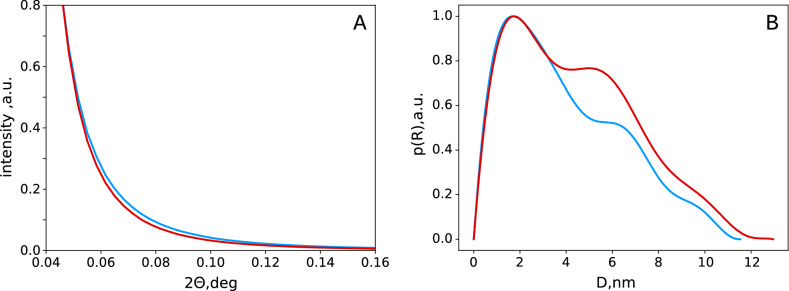
Synchrotron X-ray small-angle ($1.54\,\AA {}$ in transmission mode) scattering curves (**A**), and the result of their approximation with the model of the infinite cylinders (**B**). The results presented for the initial (blue curve) and mercerized (red curve) cotton linter cellulose.

The approximation of the small-angle X-ray scattering (SAXS) curves (Fig. 3A) of synchrotron radiation by the model of infinite cylinders revealed fibers having radii of $\sim 1.7$, 6.1, and $9.6\,\hbox {nm}$ (Fig. 3B). Despite the relative crudeness of the approach, the first and the most pronounced peak could be attributed to the nanofibrils^7,47^. The second and third ones, probably, could be attributed to the microfibrils and their bundles^12,13^. Unlike the WAXS spectra, mercerization has only a little or no effect on the distribution of the fibers over diameters (Fig. 3). The shape and position of the main peak (1.7 nm) preserved upon mercerization, indicating the preservation of the nanofibrils’ structure. Herewith, the middle peak amplitude (6.1 nm) increased significantly, and its maximum shifted to 5.2 nm, while the third peak (9.6 nm) almost disappears. We tend to attribute these effects with the changes in the internal structure of microfibrils, which lose some of the low-molecular-weight components upon alkaline treatment^20–22^. This proposition explains the decrease in the thickness of microfibrils observed experimentally.

### IR spectra

**Figure 4:**
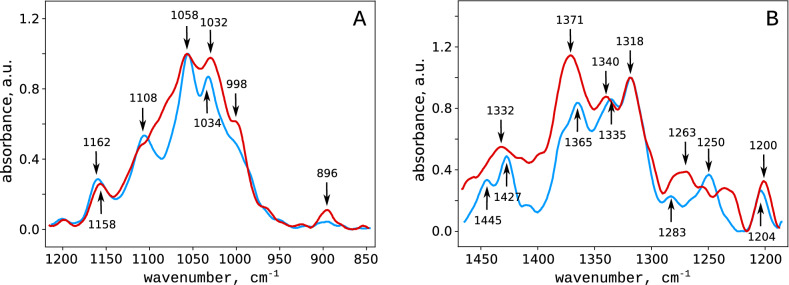
Infrared absorption spectra of the initial (blue curves) and mercerized (red curves) hardwood cellulose. For clarity, the spectra were spitted into two ranges: 1200–850 (**A**) and 1450–1200 $\hbox {cm}^{-1}$ (**B**).

Mercerization causes significant changes in the IR spectra of cellulose^48–50^. First of all, the spectrum of the mercerized cellulose contained the band at $896\,\hbox {cm}^ {-1}$ (Fig. 4A), which corresponds to the vibrations of the $\beta$-glycosidic (C–O–C) bond^51,52^. The band corresponding to the asymmetric vibrations of C–O–C bonds^51,52^ shifted from 1162 to 1158 $\hbox {cm}^{-1}$. The spectrum changed substantially in the region of 1058–1034 $\hbox {cm}^{-1}$, corresponding to C–O bonds’ vibrations in the CHOH-group^51^. Two narrow peaks at 1427 and 1445 $\hbox {cm}^{-1}$ (Fig. 4B) corresponding to the bending vibrations of the C–O–H groups^51^ disappeared, and the corresponding region of the spectrum broadened and lost the fine structure. The peak at 1108 $\hbox {cm}^{-1}$, usually associated with asymmetric valence vibrations of the pyranose rings^51^ also disappeared.

## Discussion

### Interpretation of the experimental results

The alkaline treatment causes the formation of cellulose alcoholates, which affects the geometry of the glucopyranose rings^27^ and explains the disappearance of the corresponding peak in the IR spectrum at 1108 $\hbox {cm}^{-1}$ (Fig. 4). At the same time, the intensity of the signal corresponding to the stretching of the $\beta$-glycosidic bonds increases, which is typical for amorphous regions of cellulose^50,52^. In the mercerized cellulose, we observed broadening of the IR peaks corresponding to the motion of the atoms involved in the hydrogen bonding (1058, 1034, 1427, and 1445 $\hbox {cm}^{-1}$). Therefore, in the mercerized cellulose, the hydrogen bond net seems to be partially disrupted, while these interactions stabilize the structure of the nanofibrils in the native cellulose^53^.

In the X-ray diffraction patterns of mercerized cellulose (Fig. 2), peaks shift toward the smaller angles. In the unmercerized cellulose, the main peaks were placed at 4.0 and 5.6–$5.7\,\AA {}$, for (200) and $(1{\bar{1}}0)/(110)$, respectively, while in the mercerized cellulose — at 4.1, 4.4, and 7.3 Å, for (020), (110), and $(1{\bar{1}}0)$, correspondingly. The increase of the lattice spacings (irrespective of their Miller indexes) while maintaining the fibers’ morphological structure can be interpreted as the swelling of the microcrystalline regions^3^. The latter can occur only in the course of the nanofibrils’ untwisting as a whole^3^ due to their helical structure^7,54^. The swelling of the mercerized cellulose fibers as a whole was confirmed microscopically and amounted to $\sim 5\%$.

At first glance, the results of the approximation of the SAXS curves by the model of infinite cylinders (Fig. 3) contradicts the conclusions made from cellulose swelling. Indeed, the nanofibrils’ diameter remained the same, while some larger structures (presumably, microfibrils) become thinner. However, the model used implies some cylindrical objects with a more or less clear boundary to be present in the system. Many chemical processes in cellulose, in particular nitration^3^ or acetylation^55^, start on the surface of microcrystalline regions and then spread inward. Therefore, the fibrils’ surface should be disordered to a greater extent than the internal areas, which should cause a decrease in the effective size of the ordered core of these elements. This allowed us to propose that in the fiber diameter distributions (Fig. 3), we observed the signals from the inner and dense regions of the fibrils only, which explains the observed decrease in their diameters.

### Restrictions on the reorganization of the cellulose matrix

It is generally accepted that the macromolecules have the antiparallel orientation in cellulose II^20,27–30^. Since in the native cellulose I, the chains are arranged in a parallel manner^4,16–19^, the cellulose I $\rightarrow$ cellulose II transition should be accompanied by a significant reorganization of the pulp’s structure. Indeed, the microcrystallite or nanofibril of cellulose is a dense solid body^7^, so rearrangement of its structure in the antiparallel fashion requires the disruption of the initial packing and turning some chains upside-down. The native cellulose chains are packed into the helical nanofibrils^7^, the latter twists into the microfibrils, which also form helical bundles^12,13^. Therefore, the disruption of the structure of the microcrystallites seems to be significantly hindered by the friction forces and sterical restrictions from the neighboring elements of the pulp^3^. This problem is not actively discussed, while it seems to be of crucial importance for understanding the processes in cellulose pulp.

In the mercerized cellulose, the antiparallel arrangement could be achieved if some of the chains in the microcrystallite make a U-turn. This possibility seems to be doubtful, as the morphology of the cellulose does not change upon mercerization (Fig. 1), while the swelling and the increase in the bulk volume in the cellulose fibers are negligible. Hence, the macromolecules seem to preserve their spatial positions. The degree of polymerization in our experiments was about 1500, so the macromolecules’ length would be about 750 nm. The helical pitch of the nanofibrils is about 100 nm^3,7,54^. Therefore, in the nanofibrils, the macromolecules wrap around each other several times. Thus, the U-turn of the macromolecule requires the full untwisting of the nanofibril. Otherwise, the chains would claw each other, and no U-turn would be possible. The untwisting is a rather slow process, which takes time of at least half-hour^3,55^; however, upon alkali treatment, the cellulose diffractogram entirely changes for less than 20 min^56^.

There is another theoretical observation against the antiparallel packing of mercerized cellulose. Due to the nanofibrils’ helical structure, the U-turning of the macromolecule requires its separating from other macromolecules in the nanofibril and becoming able to move independently from them. Following the general statistical laws, the lone cellulose macromolecules would fold into the Gaussian clews, which were observed experimentally as spherical beads on the surface of the regenerated cellulose^44^, and were not found in mercerized cellulose. The Gaussian clews should then unfold, and the “linearized” macromolecules should place the same position as in the native cellulose but in an upside-down orientation already.

Let the cellulose macromolecule is composed of *N* approximately isometric monomers with a characteristic linear size *d*. Being elongated, the macromolecule would occupy a volume $V \sim N \cdot d^3$. Let *l* to be the length of the Kuhn segment of the cellulose macromolecule, expressed in the number of monomer units. The free macromolecule of cellulose would fold into an isometric Gaussian clew, consisting of $N^* \sim N/l$ units, while the characteristic linear size of the unit $d^*$ would be $\sim l \cdot d$. Therefore, the characteristic linear size of the entire Gaussian clew $L^*$ would be $\sim d^* \sqrt{N^*} \sim d \sqrt{N \cdot l}$, while its volume $V^*$ would be $\sim (L^*)^3 \sim d^3 \sqrt{(N \cdot l)^3}$. Thus, the volume of the lone macromolecule would increase by $V^*/V \sim \sqrt{N \cdot l^3}$ times. Even considering the macromolecule to be extremely flexible and assuming the length of the Kuhn segment *l* to be extremely small and equal to unity, we obtain for the hardwood and cotton cellulose the ratio of the volumes of about 35–39 and 37–41, respectively. For the flax pulp, this ratio significantly exceeds 100, although the mercerization of this raw proceeds just as for cellulose from other sources. According to our estimates, the increase in the bulk volume upon mercerization was only 5–10%, which is far not enough for the Gaussian clew formation, according to the assessments above. Therefore, the U-turning of the cellulose chains requires an order of magnitude increase of the bulk volume in the pulp, which could be achieved in the full dissolution, but not swelling. Following this conclusion, we should also explicitly outline that, to our knowledge, there are no fundamental restrictions on the U-turning of the chains in the course of the cellulose regeneration from the solutions.

The alternative and a quite reasonable hypothesis imply that the antiparallel packing of the chains originates on the border between two initially antiparallel microcrystallites. The possibility of such mutual orientation of nanofibrils was demonstrated by silver staining of both ends of the bundle of the microcrystallites in mercerized ramie cellulose^35^. On the contrary, in the mercerized cotton linter, the silver staining was always observed only on one of the ends of the lone micrcrystallite^34^. Nevertheless, forming of the antiparallel packing in the interface of two adjacent nanofibrils faces the same problem as the “U-turning” hypothesis. Indeed, each chain is wrapped around the helical nanofibril seven times, on average. Accordingly, the cellulose chains on the nanofibril surface cannot fully penetrate the adjacent nanofibril, as they do not even contact the neighboring microcrystallite throughout its length. Therefore, both adjacent microcrystallites have to be untwisted and dissociated before the chains’ mutual penetration. The full untwisting and dissociation of the nanofibrils upon mercerization seems to be low probable due to the high rate of the diffractogram changing^56^, insufficient growth of the bulk volume, and cellulose morphology preservation. Therefore, the antiparallel packing can appear only among the cellulose chains located on the surfaces of the adjacent microcrystallites.

The nanofibrils’ cross-section size vary from $\sim 2.5\,\hbox {nm}$ in softwood to $\sim 9\,\hbox {nm}$ in cotton^7,10^. Therefore, the share of the fibrils capable of mutual penetration does not exceed one half even in the thinnest spruce elementary fibrils, while in cotton this portion would be drastically lower. Therefore, even in the densely packed bundle of microcrystallites, only one half of the chains can theoretically form the antiparallel structure. Moreover, given that only 15% of the bundles of microcrystallites were silver-stained at both ends^35^, the portion of the cellulose chains that can participate in the antiparallel packing would be far less than 10%. However, the WAXS data (Fig. 2) denotes that all crystallites convert into cellulose II upon alkaline treatment. Indeed, the absence of the peak (200), for example, in the cellulose II diffractograms (Fig. 2) directly implies that there is no cellulose I in the samples of the mercerized cellulose. The intensity of the halo from the amorphous regions also did not increase (Fig. 2), which, in its turn, implies that the cellulose I has fully transformed into the cellulose II, rather than became amorphous, including the core regions of the microcrystallites.

## Conclusion

Summarizing all of the above, we can state that the lack in the bulk volume in the swelled cellulose pulp, together with the superhelical nature of the native cellulose, indicates the impossibility of U-turning of the macromolecules upon mercerization. This conclusion is strongly supported by the preservation of the morphology of the cellulose fibers. On the other hand, all of the mentioned restrictions disappear in cellulose dissolution as the bulk volume grows to infinity and the initial packing breaks down completely. Upon the subsequent regeneration, cellulose chains find some alternative packing, usually having little common with the initial one. Therefore, the regenerated cellulose II can have antiparallel packing.

The hypothesis on the antiparallel packing of the cellulose chains in the interface of the adjacent antiparallel microcrystallites does not imply the U-turning of the macromolecules. However, nanofibrils’ helical structure makes them tight bodies and prevents mixing the chains with different orientations. The latter dramatically decreases the amount of the chains capable of participating in antiparallel packing forming, while the WAXS data indicates all of cellulose I has transformed to cellulose II, and there is not so much amorphous phase in mercerized cellulose.

The alternative to the antiparallel structure of the mercerized cellulose could be some parallel packing, having similar lattice parameters. There is at least one example of such parallel packing (p2 *gt/gt*) described in the literature^28,39^ allowing overcoming all of the difficulties described in this work. It is also noteworthy that the molecular dynamic simulation demonstrated the possibility of the transition between II p2 *gt*/*gt* and I$\beta$,
